# Optimizing the Follow‐Up Interval After Successful Cold Knife Conization of CIN3: A 10‐Year Retrospective Cohort Study

**DOI:** 10.1002/cam4.70825

**Published:** 2025-03-29

**Authors:** Kun Fu, Kelsang Yangzom, Lucia Li, Lisha Wu, Yu Zhang

**Affiliations:** ^1^ Department of Gynecology Xiangya Hospital, Central South University Changsha China; ^2^ Gynecological Oncology Research and Engineering Center of Hunan Province Changsha China; ^3^ National Clinical Research Center for Geriatric Disorders Xiangya Hospital, Central South University Changsha China; ^4^ Institute of Medical Sciences Xiangya Hospital, Central South University Changsha China

**Keywords:** cervical intraepithelial neoplasia grade 3, cold knife conization, HPV persistence, negative margin

## Abstract

**Background:**

This study was conducted to identify the risk of residual or recurrent high‐grade squamous intraepithelial lesions or worse (HSIL+) in patients with successful conization and to develop a customized management strategy.

**Methods:**

This retrospective study included 939 patients who underwent cold knife conization (CKC) for cervical intraepithelial neoplasia 3 at a hospital in China between January 1, 2013 and December 31, 2020. Demographic characteristics and test results were obtained before and 6, 12, and 24 months after CKC and annually thereafter. Human papillomavirus (HPV) persistence was defined as HPV positive at both 6 and 12 months after CKC, and the primary endpoint was residual or recurrent HSIL+ after CKC.

**Results:**

The mean follow‐up period was 68.8 months. In total, 61 (6.5%) patients had HPV persistence, and 19 (2.0%) had residual or recurrent HSIL+. The risk of residual or recurrent HSIL+ was increased in patients with HPV infection at 6 months (hazard ratio [HR], 84.6; 95% confidence interval [CI], 11.2–641) and 12 months (HR, 214; 95% CI, 28.1–1625) after CKC, and HPV persistence after CKC (HR, 244; 95% CI, 32.2–1854). Comparing two different colposcopic referral criteria for HPV persistence and HPV positive 6 months post‐CKC, substantially fewer colposcopies were performed per case of residual or recurrent HSIL+ detected in patients with HPV persistence after CKC (3.39 vs. 8.28).

**Conclusions:**

The risk of residual or recurrent HSIL+ was higher in patients with HPV persistence after CKC. In patients with negative margins, extending the follow‐up interval to 12 months may reduce the number of HPV tests and colposcopy referral rates while maintaining HSIL+ detection.

## Graphical abstract

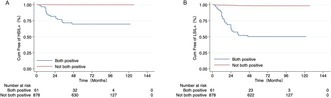

## 1 Introduction

Screening and treatment of precancerous lesions of the cervix can substantially reduce the risk of developing cervical cancer. High‐grade squamous intraepithelial lesions (HSIL) of the cervix, including cervical intraepithelial neoplasia (CIN)2 and CIN3, are precancerous lesions [1].

In patients with CIN3, conization, including loop electrosurgical excision procedure (LEEP), large loop excision of the transformation zone, cold knife conization (CKC), or laser cone biopsy, is recommended, and observation is unacceptable [2]. The margin of conization is categorized as positive or negative based on the presence or absence of a CIN lesion, respectively, and the later one is regarded as successful conization [3]. A positive margin is an independent risk factor for residual or recurrent lesions after conization [4, 5, 6], and patients with positive margins should be closely monitored, and surgery should be repeated if necessary [2].

Patients with a negative margin are recommended to undergo human papillomavirus (HPV) testing 6 months after surgery [7]. If three consecutive HPV tests at 12‐month intervals are negative, the frequency of HPV testing can be extended to a 3‐year interval, and at least 25 years of follow‐up is recommended [2, 7]. Individuals with HPV infection should be referred for colposcopy. The prevalence of HPV persistence decreases over time from 14.3%–50.0% at 6 months after conization to 2.2%–22.4% at 12 months after conization [8, 9, 10]. This result in a high colposcopic referral rate for HPV positive patients 6 months after conization. Furthermore, given the small percentage of cervical cancers [11, 12] found after conization, it is necessary to identify risk factors for HSIL+ residual or recurrence after successful conization (with negative margin) and to provide clinical indications for colposcopic referral of such patients. Two studies with a small sample size and short follow‐up time have found that patients with HPV16‐positive lesions or undergoing LEEP are more likely to have residual or recurrent HSIL [13, 14]. However, there were few long‐term follow‐up studies. This study included patients who underwent CKC for CIN3 with negative margins with a follow‐up period of up to 10 years; therefore, the results provide a source reference for follow‐up of patients with negative margins.

## 2 Methods

### 2.1 Study Design

In this retrospective cohort study, we reviewed the records of 2257 patients treated by CKC at our hospital between January 1, 2013, and December 31, 2020. Patients included in this study had CIN3 histologically confirmed by colposcopic biopsy before CKC. Patients were excluded if they met any of the following criteria: (1) cervical cancer based on the pathological results of CKC; (2) positive margins on CKC; (3) secondary conization or hysterectomy within 12 months after CKC; (4) less than 24 months of follow‐up or no testing for HPV within 12 months after CKC; and (5) positive for HPV for 12 months but had not undergone a biopsy. Demographic characteristics including name, age, ID, smoking history, reproductive history, and history of infectious disease before CKC were recorded. All enrolled patients provided verbal and written consent to participate in the study. The hospital's institutional review board approved the study protocol (approval number: 2018121117).

### 2.2 Diagnostic Strategies

The diagnosis and screening strategies were based on those used in a previous study [15]. The pathological diagnosis of the CKC specimens was determined based on the most serious lesion present, according to the World Health Organization classification of tumors of female reproductive organs [1]. CIN1 lesions were classified as LSIL, and CIN2 and CIN3 lesions were classified as HSIL. Pathological diagnoses of all tissues were assessed and confirmed separately by two experienced pathologists.

### 2.3 Follow‐Up

According to the guidelines issued by the Chinese Society for Colposcopy and Cervical Pathology, patients underwent HPV based or combined HPV and cytology testing at 6, 12, and 24 months after conization, and annually thereafter. Colposcopy was performed in patients with positive HPV results or abnormal cytological findings. If cervical dysplasia was suspected, usually two to four biopsies were performed during each colposcopy procedure. Biopsy was not recommended in patients with cytology results less than HSIL, no evidence of HPV 16/18 infection, and normal appearance on colposcopy. All colposcopies were performed by well‐trained and experienced physicians. Patients with HPV negative results, normal cytology, and appearance on colposcopy were defined as free of lesions. If LSIL+ lesions were detected, the follow‐up outcome was defined according to pathological findings. The primary endpoint was residual or recurrent HSIL or worse (HSIL+) detected during follow‐up. The secondary endpoint was residual or recurrent LSIL or worse (LSIL+) detected during follow‐up. HPV persistence was defined as persistently positive HPV at both 6 and 12 months or more after CKC. The follow‐up time was from baseline to the date of the study endpoint or July 31, 2023, whichever occurred first.

### 2.4 Statistical Analysis

The distribution of categorical variables among patients with and without residual or recurrent HSIL+ was compared using the log‐rank test. Differences were considered statistically significant if the two‐sided P value was < 0.05. The cumulative proportion without residual or recurrent HSIL+ was displayed using Kaplan–Meier curves, and the differences were compared using log‐rank tests. Cox proportional hazards regression was used to calculate hazard ratios and 95% confidence intervals (CIs) to estimate the risk of residual or recurrent HSIL+ according to different predictors. All data analyses were performed using SPSS, version 27.0 (IBM Corp., Armonk, NY, USA), and Stata, version 17.0 (StataCorp, College Station, TX, USA).

## 3 Results

### 3.1 Patient Characteristics

**FIGURE 1:**
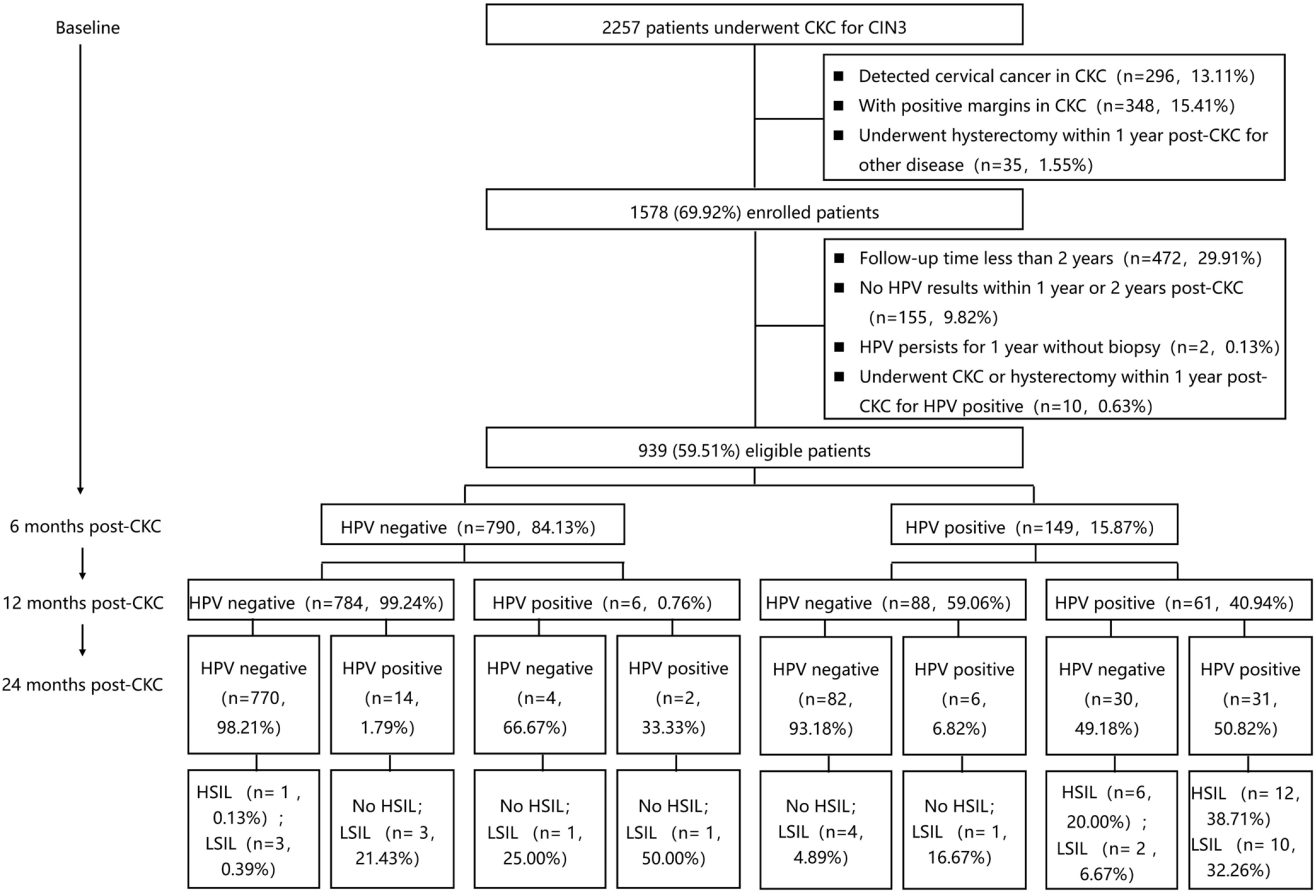
The flow of patients. CKC, cold knife conization; HPV, human papillomavirus; HSIL, high‐grade squamous intraepithelial lesions; LSIL, low‐grade squamous intraepithelial lesions.

Of 2257 patients who underwent CKC for CIN3, 296 (13.11%) patients had cervical cancer detected on CKC, 348 (15.41%) patients had positive margins on CKC, and 35 (1.55%) patients underwent hysterectomy within 12 months after CKC for other disease were excluded. Of the remaining 1578 (69.92%) patients, 472 (29.91%) patients who were followed up for less than 24 months, 155 (9.82%) patients who did not have HPV test results within 12 or 24 months after CKC, 2 (0.13%) patients with persistent HPV infection for 12 months who did not undergo biopsy, and 10 (0.63%) patients who underwent CKC or hysterectomy within 1 year post‐CKC for HPV positive were subsequently eliminated. After these exclusions, 939 patients were included in the final study cohort (Figure 1). The mean age at surgery was 40.4 years (range 20–68 years).

### 3.2 Patient Follow‐Up

**FIGURE 2:**
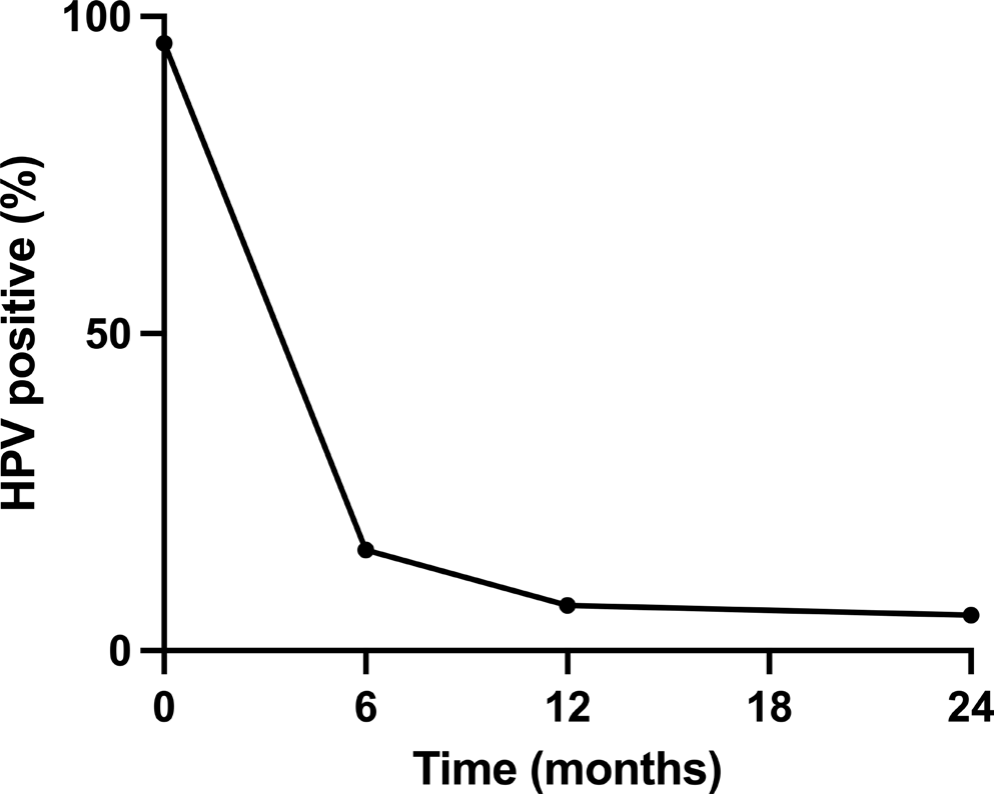
The rates and patterns of HPV clearance after CKC. CKC, cold knife conization, HPV, human papillomavirus.

During follow‐up, 895 patients (95.31%) did not have cervical lesions detected, 25 (2.66%) were diagnosed with LSIL, and 19 (2.02%) were diagnosed with HSIL+. The patient characteristics are shown in Table 1. The mean follow‐up time was 68.82 months (range 30–127 months). HPV was detected in 15.87% (149/939) at 6 months, 7.14% (67/939) at 12 months, 5.64% (53/939) at 24 months, and 1.70% (18/939) more than 24 months after CKC (Figure 2). A total of 61 patients had HPV persistence after CKC.

**TABLE 1:** Baseline characteristics.

Characteristic	No lesions (*n* = 895, 95.31%)	LSIL (*n* = 25, 2.66%)	HSIL (*n* = 19, 2.02%)	Total (*n* = 939)
Age, years	40.08 ± 8.33	45.44 ± 10.91	46.89 ± 8.73	40.36 ± 8.50
Menstrual status				
Premenopause (%)	796 (96.95)	15 (1.83)	10 (1.22)	821 (100)
Postmenopause (%)	99 (83.90)	10 (8.47)	9 (7.63)	118 (100)
Number of pregnancies				
≥ 4 (%)	376 (94.71)	12 (3.02)	9 (2.27)	397 (100)
< 4 (%)	519 (95.76)	13 (2.40)	10 (1.85)	542 (100)
Number of deliveries				
≥ 2 (%)	394 (93.59)	13 (3.09)	14 (3.33)	421 (100)
< 2 (%)	501 (96.72)	12 (2.32)	5 (0.97)	518 (100)
HPV				
Negative (%)	34 (94.44)	1 (2.78)	1 (2.78)	36 (100)
Positive (%)	778 (95.58)	18 (2.21)	18 (2.21)	814 (100)
Unknown (%)	83 (93.26)	6 (6.74)	0 (0)	89 (100)
Cytology pre‐CKC				
Normal (%)	172 (94.51)	4 (2.20)	6 (3.30)	182 (100)
ASCUS+ (%)	518 (95.57)	16 (2.95)	8 (1.48)	542 (100)
Unknown (%)	205 (95.35)	5 (2.33)	5 (2.33)	215 (100)
Glandular involvement				
No (%)	198 (93.40)	5 (2.36)	9 (4.25)	212 (100)
Yes (%)	654 (96.18)	19 (2.79)	7 (1.03)	680 (100)
Unknown (%)	43 (91.49)	1 (2.13)	3 (6.38)	47 (100)

Abbreviations: ASCUS, atypical squamous cells of undetermined significance; CKC, cold knife conization; HPV, human papillomavirus; HSIL, high‐grade squamous intraepithelial lesions; LSIL, low‐grade squamous intraepithelial lesions.

### 3.3 Risk Factors for Residual or Recurrent Lesions After Conization

**FIGURE 3:**
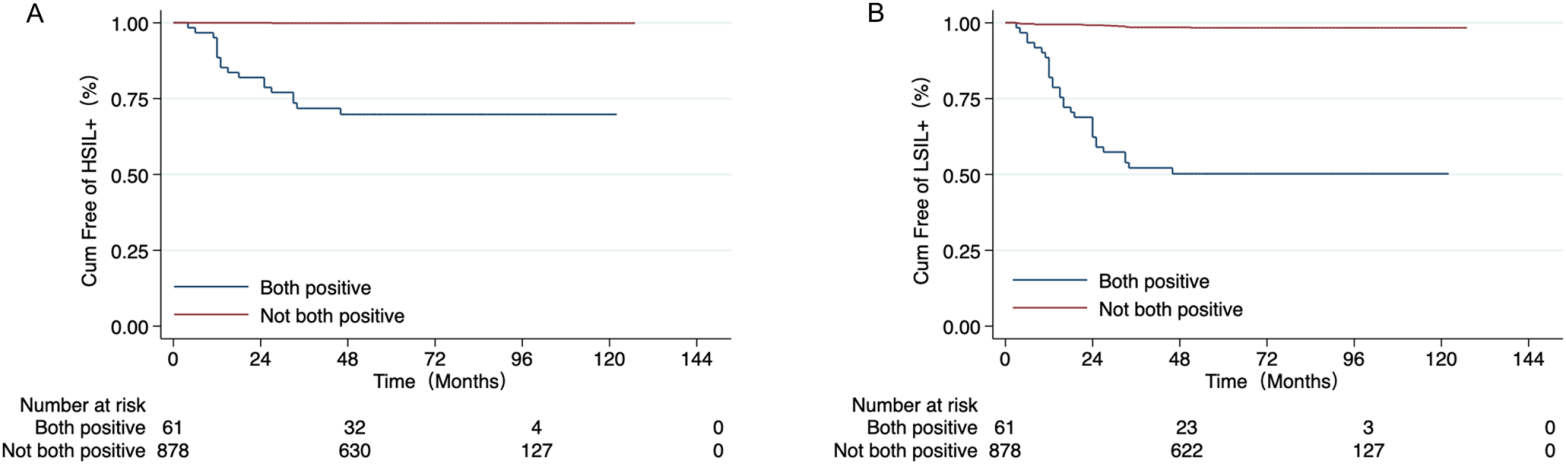
The association between HPV status and recurrent or residual lesions. (A) Kaplan–Meier plot of cumulative percentage of patients free of residual or recurrent HSIL+ according to HPV status at 6 months or 12 months post‐CKC. (B) Kaplan–Meier plot of cumulative percentage of patients free of residual or recurrent LSIL+ according to HPV status at 6 months or 12 months post‐CKC. CKC, cold knife conization; Cum, cumulative; HPV, human papillomavirus; HSIL+, high‐grade squamous intraepithelial lesions or worse; LSIL+, low‐grade squamous intraepithelial lesions or worse.

To identify the risk factors for residual or recurrent lesions after CKC, patients were grouped according to age (< 40/≥ 40 years), HPV infection, glandular involvement, and menstrual status. We found no association between these factors and the risk of residual or recurrent HSIL+ after CKC. After adjusting for other factors, the risk of residual or recurrent HSIL+ was increased in patients with HPV infection at 6 months (hazard ratio [HR], 84.6; 95% CI, 11.2–641), and 12 months (HR, 214; 95% CI, 28.1–1625), and in those with HPV persistence (HR, 244; 95% CI, 32.2–1854) after CKC (Table 2). Similarly, after adjusting for other factors, the risk of residual or recurrent LSIL+ was increased in patients with HPV infection at 6 months (HR, 19.2; 95% CI, 9.13–40.5), and 12 months (HR, 36.1; 95% CI, 18.1–71.7), and in those with HPV persistence (HR, 33.0; 95% CI, 17.1–63.7) after CKC (Table 3). Furthermore, patients with persistent HPV infection had an increased risk of residual or recurrent HSIL+ (Figure 3A) or LSIL+ (Figure 3B) and a shorter time to onset.

**TABLE 2:** Cox regression analysis of the factors associated with residual or recurrent HSIL+ after CKC.

	Adjusted HR^a^	95% CI	P value
HSIL			
Age			
< 40	Ref		
≥ 40	3.21	0.83–12.46	0.091**
Menstrual status			
Premenopause	Ref		
Postmenopause	1.87	0.68–5.19	0.227
Glandular involvement			
No	Ref		
Yes	1.09	0.63–1.90	0.748
HPV results at 6 months after CKC			
Negative	Ref		
Positive	84.59^b^	11.17–640.56^b^	< 0.001***
HPV results at 12 months after CKC			
Negative	Ref		
Positive	213.81^b^	28.14–1624.75^b^	< 0.001***
Combined HPV results at 6 and 12 months after CKC			
Not both positive	Ref		
Both positive	244.38^b^	32.20–1854.49^b^	< 0.001***

Abbreviations: CI, confidence interval; CKC, cold knife conization; HPV, human papillomavirus; HR, hazard ratio; HSIL+, high‐grade squamous intraepithelial lesions or worse.

^a^ Hazard ratios were adjusted for other factors in the analysis.

^b^ Statistically significant HR and CI.**P < 0.01, ***P < 0.001.

**TABLE 3:** Cox regression analysis of the factors associated with residual or recurrent LSIL+ after CKC.

	Adjusted HR^a^	95% CI	*p*
LSIL+			
Age			
< 40	Ref		
≥ 40	1.34	0.02–2.98	0.476
Menstrual status			
Premenopause	Ref		
Postmenopause	3.04	1.46–6.30	0.003**
Glandular involvement			
No	Ref		
Yes	1.15	0.80–1.67	0.451
HPV results at 6 months after CKC			
Negative	Ref		
Positive	19.24^b^	9.13–40.52^b^	< 0.001***
HPV results at 12 months after CKC			
Negative	Ref		
Positive	36.05^b^	18.12–71.70^b^	< 0.001***
Combined HPV results at 6 and 12 months after CKC			
Not both positive	Ref		
Both positive	32.97^b^	17.06–63.74^b^	< 0.001***

Abbreviations: CI, confidence interval; CKC, cold knife conization; HPV, human papillomavirus; HR, hazard ratio; LSIL+, low‐grade squamous intraepithelial lesions or worse.

^a^ Hazard ratios were adjusted for other factors in the analysis.

^b^ Statistically significant HR and CI.**P < 0.01, ***P < 0.001.

### 3.4 Efficiency of Different Colposcopy Referral Criteria

Patients with HPV infection at 6 months after CKC were referred for colposcopy according to the current guidelines. To determine the role of subsequent colposcopy in the detection of HSIL+ or LSIL+ in the presence of persistent HPV infection, and to reduce the rate of colposcopy referrals, we compared the efficiency of colposcopy referral in those with HPV infection at 6 months, at 12 months, and with persistent HPV infection (at both 6 and 12 months) after CKC.

In the diagnosis of HSIL+, the specificity and area under the curve (AUC) of colposcopy referral based on HPV persistence and a 12‐month HPV positive result post‐CKC were higher than those based on a 6‐month HPV positive result post‐CKC, and the sensitivity values were similar. Colposcopy referral of those with a 12‐month HPV positive result and HPV persistence post‐CKC detected all recurrent HSIL+ lesions. If only those with persistently positive HPV were referred for colposcopy, the number of referrals for colposcopy was 61, and 3.39 colposcopies were required for each HSIL+ detected, which was lower than the 8.28 colposcopies required for each HSIL+ detected using a strategy of colposcopy referral of those who were HPV positive at 6 months after CKC (Table 4).

**TABLE 4:** Colposcopy referral and HSIL+ or LSIL+ detection for three different colposcopic referral criteria.

Colposcopic referral criteria	Sensitivity (95% CI)	Specificity (95% CI)	AUC (95% CI)	No. of colposcopies	No. of lesions detected	No. of Colposcopies per lesions Detected
HSIL+ detection						
HPV positive 6 months post‐CKC	94.74% (75.36%–99.73%)	85.76% (83.35%–87.87%)	0.903 (0.842–0.963)	149	18	8.28
HPV positive 12 months post‐CKC	94.74% (75.36%–99.73%)	94.67% (93.03%–95.95%)	0.947 (0.889–1.000)	67	18	3.72
HPV persistence post‐CKC	94.74% (75.36%–99.73%)	95.33% (93.76%–96.51%)	0.950 (0.892–1.000)	61	18	3.39
LSIL+ detection						
HPV positive 6 months post‐CKC	79.55% (65.50%–88.85%)	87.26% (84.92%–89.29%)	0.834 (0.764–0.904)	149	35	4.26
HPV positive 12 months post‐CKC	72.73% (58.15%–83.65%)	96.09% (94.61%–97.17%)	0.844 (0.765–0.923)	67	32	2.09
HPV persistence post‐CKC	68.18% (53.44%–80.00%)	96.54% (95.13%–97.55%)	0.824 (0.740–0.907)	61	30	2.03

Abbreviations: CI, confidence interval; CKC, cold knife conization; HPV, human papillomavirus; HSIL+, high‐grade squamous intraepithelial lesions or worse; LSIL+, low‐grade squamous intraepithelial lesions or worse.

In the diagnosis of LSIL+, compared with a colposcopy referral strategy based on a positive HPV result 6 months post‐CKC, HPV persistence and a 12‐month HPV positive result post‐CKC had higher specificity, lower sensitivity, and comparable AUC. If only those with persistently positive HPV were referred for colposcopy, the number of referrals for colposcopy was 61, and 2.03 colposcopies were required for each LSIL+ detected, which was lower than the 4.26 colposcopies required for each LSIL+ detected using a strategy of colposcopy referral of those who were HPV positive at 6 months after CKC; however, not all LSIL lesions were detected (Table 4).

## 4 Discussion

This retrospective cohort study followed up women who underwent CKC and had negative margins on conization for up to 10 years. The risk of residual or recurrent HSIL+ was higher in patients with HPV persistence for 12 months compared with HPV positive at 6 months after CKC. In patients with negative margins, extending the follow‐up interval to 12 months may reduce the number of HPV tests and colposcopy referral rates while maintaining HSIL+ detection.

According to literature reports, positive margin is a high‐risk factor for residual or recurrent HSIL+ after cervical conization and has been widely studied and recommended for close surveillance or reoperation [2, 15, 16]. However, few studies have focused on residual or recurrent HSIL+ in patients with negative margins, which are approximately four times more common than positive margins, and guidelines do not make a distinction [4, 15]. The Chinese Expert Consensus on the Management of High‐grade Cervical Intraepithelial Lesions recommends HPV testing at 6 months after CKC, regardless of the margin status of the excisional specimen or whether previous HPV tests were positive, and that colposcopy and appropriate biopsies should be performed in patients with positive HPV results. These recommendations are the same as those in the 2019 ASCCP Risk‐Based Management Consensus Guidelines for Abnormal Cervical Cancer Screening Tests and Cancer Precursors [2, 7]. Although most patients with HPV positive results reverted to negative within 6 months after conization, many patients test HPV positive at 6 months after CKC. In our study, HPV infection was detected in 15.87% of patients at 6 months and 7.14% at 12 months, consistent with the results of previous studies [8, 9, 10]. However, only 12.08% of patients with HPV positive at 6 months after CKC had residual or recurrent HSIL+. Follow‐up at 6‐month intervals according to the guidelines would lead to overtesting, and colposcopy referral for all patients with HPV infection is unnecessary. Therefore, specific guidelines are required for the management of patients with negative incisal margins.

Two studies have attempted to identify the risk factors for residual or recurrent HSIL+ after successful conization (with negative margins) and provide clinical indications. Akiko et al. [13] included 211 patients who were followed up for 3 years and found that 23 patients were HPV positive after surgery, and those who were positive for HPV16 before surgery were more likely to have persistent HPV infection. However, the sample size was small and the follow‐up time was relatively short; additionally, most residual or recurrent HSIL were detected within 4 years after surgery [17, 18]. Lili et al. [14] included 804 patients who underwent LEEP or CKC for HSIL and found that 1.1% (95% CI: 0.5%–2.2%) of the patients had residual or recurrent HSIL, consistent with our result (2.02%). However, they only compared the risk of residual or recurrent HSIL between patients undergoing LEEP and CKC and did not consider other factors.

In this study, we analyzed the association of age, menstrual status, glandular involvement, and HPV results after CKC with residual or recurrent HSIL+ or LSIL+. We found that once HPV infection was detected, the risk of residual or recurrent HSIL+ or LSIL+ was substantially higher, particularly in patients with HPV positivity at 12 months after CKC or HPV persistence for 12 months. The risk of developing residual or recurrent LSIL+ was higher in postmenopausal patients than in premenopausal patients. These results provide an evidence base for delaying follow‐up and reducing the frequency of HPV testing and colposcopy referral after CKC in patients with negative margins. In patients with HPV persistence for 12 months or more, age and menstrual condition were not associated with the risk of residual or recurrent HSIL+, possibly because almost half of the patients with HPV persistence were older than 45 years or had undergone menopause.

Finally, we compared the diagnostic accuracy and colposcopic referral rate of patients with residual or recurrent HSIL+ or LSIL+ using three colposcopic referral criteria (HPV positive results 6 months after CKC, HPV positive results 12 months after CKC, and HPV persistence after CKC). Substantially fewer colposcopies were performed per case of residual or recurrent HSIL+ detected in patients with HPV persistence after CKC (3.39 vs. 8.28) with AUC being higher (0.950 vs. 0.903). Limiting referral for colposcopy to patients with HPV positivity 12 months after CKC or HPV persistence could reduce the colposcopy referral rate while maintaining the sensitivity for diagnosing residual or recurrent HSIL+ and LSIL+. However, the sensitivity of the diagnosis of residual or recurrent LSIL+ and the AUC were lower when HPV positivity 12 months after CKC or HPV persistence was used as the referral criterion, possibly because some LSIL lesions spontaneously regressed and transient HPV infection cleared spontaneously after CKC. The colposcopy physicians performing the procedure were well trained and experienced; therefore, we assume that each colposcopy procedure was performed correctly and that lesion detection was accurate.

Strengths of this study include the large sample size and prolonged follow‐up period of up to 10 years. Moreover, we analyzed the association between persistent HPV infection and residual or recurrent HSIL+ after CKC in patients with negative margins. This study provides a source of reference for the management of patients with negative margins after CKC.

This study has some limitations. Its main limitation is that it is a retrospective study. Some patients were lost to follow‐up because of inaccurate contact information and irregular follow‐up. We defined HPV persistence as the persistence of HPV infection for 12 months or more after CKC and did not consider the effect of HPV type, which helps improve risk stratification of HPV positive [19, 20]. Combined screening for HPV and cytological analysis, as well as colposcopy on HPV negative patients to confirm the absence of lesions, were not performed; therefore, diagnosis of HPV negative cervical lesions might have been missed. No factors associated with residual or recurrent HSIL+ were found in patients with persistent HPV infection, possibly owing to the small number of patients in this subgroup.

## 5 Conclusion

Less than one in ten patients with negative margins at CKC had persistent HPV infection after CKC. In patients with negative margins at CKC, HPV testing with an interval of 12 months instead of 6 months may reduce not only the rate of HPV testing but also the colposcopy referral rate without leading to an increase in missed cases of HSIL+.

## Author Contributions

**Kun Fu:** data curation (lead), formal analysis (lead), investigation (lead), methodology (lead), software (lead), writing – original draft (lead). **Kelsang Yangzom:** data curation (supporting), investigation (supporting). **Lucia Li:** software (supporting), validation (supporting). **Lisha Wu:** methodology (supporting), project administration (supporting), supervision (supporting), visualization (supporting). **Yu Zhang:** funding acquisition (lead), project administration (lead), validation (lead), visualization (lead).

## Ethics Statement

The study protocol was approved by the Xiangya hospital's institutional review board (Approval number: 2018121117).

## Conflicts of Interest

The authors declare no conflicts of interest.

## Data Availability

Data are available from the corresponding author upon reasonable request.
